# Transforming Growth Factor-Beta-Induced Protein (TGFBI)/(βig-H3): A Matrix Protein with Dual Functions in Ovarian Cancer

**DOI:** 10.3390/ijms130810461

**Published:** 2012-08-21

**Authors:** Miranda P. Ween, Martin K. Oehler, Carmela Ricciardelli

**Affiliations:** 1 Discipline of Obstetrics and Gynaecology, School of Paediatrics and Reproductive Health, Research Centre for Reproductive Health, Robinson Institute, University of Adelaide, Adelaide, South Australia, 5000, Australia; E-Mails: miranda.ween@adelaide.edu.au (M.P.W.); martin.oehler@adelaide.edu.au (M.K.O.); 2 Research Centre for Infectious Diseases, School of Molecular Biosciences, University of Adelaide, South Australia 5005, Australia; 3 Department of Gynaecological Oncology, Royal Adelaide Hospital, Adelaide, South Australia, 5000, Australia

**Keywords:** ovarian cancer, extracellular matrix, TGFBI, tumor suppressor, invasion, adhesion, metastasis

## Abstract

Transforming growth factor-beta-induced protein (TGFBI, also known as βig-H3 and keratoepithelin) is an extracellular matrix protein that plays a role in a wide range of physiological and pathological conditions including diabetes, corneal dystrophy and tumorigenesis. Many reports indicate that βig-H3 functions as a tumor suppressor. Loss of βig-H3 expression has been described in several cancers including ovarian cancer and promoter hypermethylation has been identified as an important mechanism for the silencing of the *TGFBI* gene. Our recent findings that βig-H3 is down-regulated in ovarian cancer and that high concentrations of βig-H3 can induce ovarian cancer cell death support a tumor suppressor role. However, there is also convincing data in the literature reporting a tumor-promoting role for βig-H3. We have shown βig-H3 to be abundantly expressed by peritoneal cells and increase the metastatic potential of ovarian cancer cells by promoting cell motility, invasion, and adhesion to peritoneal cells. Our findings suggest that βig-H3 has dual functions and can act both as a tumor suppressor or tumor promoter depending on the tumor microenvironment. This article reviews the current understanding of βig-H3 function in cancer cells with particular focus on ovarian cancer.

## 1. Introduction

**Figure 1:**
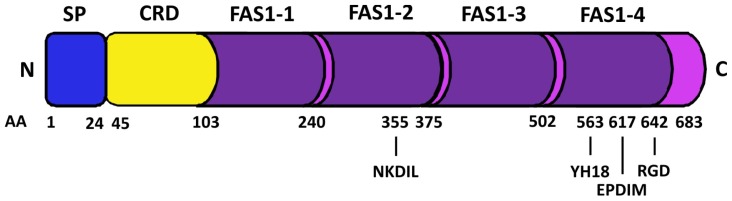
Schematic diagram of Transforming growth factor-beta-induced protein (βig-H3) protein structure. Secretory signal (SP) in the *N*-terminal cysteine rich domain (CRD), and four fasciclin-1 domains (FAS1 1–4). Position of several known integrin binding motifs, including NKDIL, YH18, EPDIM and Arg-Gly-Asp (RGD), are indicated.

βig-H3 (also known as TGFBI for protein and *TGFBI* for gene) is a transforming growth factor beta (TGFβ) inducible secreted extracellular matrix (ECM) protein. The name βig-H3 was derived from its cloning as a major TGFβ responsive gene in lung adenocarcinoma cell line A549: TGFβ induced gene human clone 3 [1]. In the literature it has also been referred to as keratoepithelin [2], collagen fibre associated protein (RGD-CAP) [3], P78/70 [4], Big-h3 [5], β-igH3 [6], and β-ig [7]. βig-H3 is comprised of 683 amino acids and its secreted form has a predicted molecular mass of 68 kDa. Two isoforms of βig-H3 at 78 and 68 kDa have been reported to date [8], both of which are encoded by a single gene, *TGFBI* [9]. βig-H3 contains an *N*-terminal secretory signal (1–24 amino acids), a cysteine rich domain, four internal repetitive fasciclin-1 domains (FAS1 1–4), integrin binding motifs in the *C*-terminus known as Arg-Gly-Asp (RGD), YH18, and EPDIM and an internal NKDIL motif [10,11] (Figure 1).

## 2. βig-H3 Regulation and Function

βig-H3 participates in many physiological processes including morphogenesis, adhesion/migration, angiogenesis, and inflammation [12]. It also has a role in reproduction [13,14] and wound healing [15,16]. A wide range of cells have been shown to induce expression of βig-H3 following treatment with TGFβ including, fibroblasts, chondrocytes, smooth muscle cells, corneal epithelial cells, and various types of cancer cells [12]. βig-H3 is regulated not only by TGFβ, but also by retinoid [17], IL-4 [15], IL-1 [18], and TNF-α [18] in various cell types. TNF-like ligand 1A can regulate the inflammatory processes in a human acute monocytic leukemia cell line (THP-1) through modulation of the βig-H3 expression via both protein kinase C and extracellular signal-regulated kinase pathways [19]. βig-H3 could also be induced in human mesenchymal stem cells by treatment with the phospholipid, lysophosphatidic acid that is enriched in the serum of cancer patients [20]. Recent evidence suggests that βig-H3 expression can also be regulated by the microRNA, miR-21 [5]. βig-H3 has been shown to trigger phosphorylation and to activate several intracellular pathways including AKT, extracellular signal-regulated kinase, focal adhesion kinase (FAK), and paxillin, thus mediating adhesion and migration of vascular smooth muscle cells through interactions with αvβ5 integrins [21].

Immunohistochemical studies show that βig-H3 is distributed in the ECM of a wide range of developing and mature tissues, including endothelial cells of human vascular tissues [22], papillary dermis [10], primary spongiosa, periosteum, and perichondrium [23]. It has also been associated with bone formation [24,25]. βig-H3 expression is induced in endothelium and stroma-derived cells in the healing cornea [7] and reactive astrocytes in rat cerebral cortex at wound sites [16].

In many cell types, βig-H3 functions as a linker protein which connects various matrix molecules to each other as well as facilitating cell-collagen interactions [4,26–28]. βig-H3 can bind to type I, II, and IV collagens as well as proteoglycans such as biglycan and decorin [28]. It has been shown that βig-H3 binds covalently to collagen VI microfibrils [27] and interacts with fibronectin [26] and various integrins [29], which are the only βig-H3 cell surface receptors identified to date (reviewed in [12]).

βig-H3 plays a role in the adhesion and migration of a wide range of cells including keratinocytes, fibroblasts, chondrocytes, osteoblasts, and endothelial cells (reviewed by [25]). Effects on adhesion are mediated through interactions with various integrins including α1β1, α3β1, αvβ3, and αvβ5 [10,30–33] via integrin binding motifs in the βig-H3 protein. These include the well characterized RGD motif in the *C*-terminus [34] as well as the NKDIL motif (amino acids 354–358) [11] and the EPDIM motif (amino acids 617–621) [11] in the second and fourth FAS-1 domains, respectively (Figure 1). The structural analysis of the NKDIL and EPDIM sequence motifs show that they can adopt a β-turn structure similar to the RGD motif to interact with integrins during adhesion [34]. Another adhesion motif shown to support αvβ5 integrin mediated adhesion of lung fibroblast MRC-5 cells [29], vascular smooth muscle cells [21], and endothelial cells [35], is the highly conserved tyrosine and histidine residues YH18 motif (amino acids 563–580) in the fourth FAS-1 domain, which is flanked by several leucine/isoleucine residues (Figure 1).

## 3. βig-H3 Roles in Disease

### 3.1. Role in Diabetes and Corneal Dystrophies

βig-H3 has been associated with a range of diseases including nephropathy [36], atherosclerosis [22], and rheumatoid arthritis [15,18], as well as corneal disorders. Its role in inflammatory disease processes is not well understood. βig-H3 expression is prominent in the kidney and increased in the urine of diabetics [37,38]. It has been suggested that combined monitoring of albumin excretion rate and urinary βig-H3 can predict the severity of diabetic nephropathy [39]. βig-H3 has been shown to induce pericyte apoptosis through its RGD motif, which may constitute an important pathogenic mechanism leading to pericyte loss in diabetes [40]. Recent studies also suggest that βig-H3 may be involved in kidney pathology associated with preeclampsia, and was detectable in the urine of these patients but not in non-preeclamptic pregnant women [41].

Mutations in the *TGFBI* gene are well characterized in a number of corneal dystrophies, which lead to the development of corneal deposits and impaired vision [42–45]. Corneal dystrophies represent the only known pathological disease associated with mutations in *TGFBI*. The mechanisms of pathogenesis are unknown but mutations in *TGFBI* may impair protein folding or βig-H3 secretion and result in the deposition and accumulation of mutant βig-H3 protein that has increased stability [46].

### 3.2. Roles in Cancer

#### 3.2.1. Role as Tumor Suppressor

Many reports indicate βig-H3 is an inhibitor of tumorigenesis and suggest that βig-H3 functions as a tumor suppressor (summarized in Table 1). Furthermore, reduced expression of βig-H3 has been observed in many tumor types. Down-regulation of βig-H3 was found to correlate highly with promoter hypermethylation in lung, prostate, and breast cancer cells. Promoter hypermethylation is considered an important mechanism involved in the silencing of the *TGFBI* gene in human cancer cells [47].

βig-H3 overexpression has been shown to markedly reduce tumorigenicity of CHO cells and lung cancer cells *in vivo* [48,50]. βig-H3 expression is markedly suppressed in asbestos- and radiation-induced tumorigenic cells, whilst ectopic expression of βig-H3 significantly suppresses tumorigenicity and progression in human bronchial epithelial cells [50–52]. βig-H3 has also been reported to mediate apoptosis through the RGD motif in CHO cells [49] and the EPDIM motif in osteosarcoma cells [57]. A recent observation by Becker *et al.* suggested that increased expression of βig-H3 suppresses neuroblastoma cell adhesion to various ECM proteins, thus inhibiting their proliferation and invasion [2]. More recent studies demonstrating that the loss of βig-H3 predisposes mice to spontaneous tumor development have provided strong *in vivo* evidence that βig-H3 functions as a tumor suppressor [58]. Mouse embryonic fibroblasts isolated from *TGFBI*^−/−^ mice displayed increased frequencies of chromosomal aberration, abnormal mitoses, and enhanced proliferation [58]. The loss of chromosomal integrity may explain the increased tumor tendency in the *TGFBI* knockout mice.

Recent studies using lung and breast cancer cell lines have also shown that βig-H3 induced adhesion to ECM proteins, but reduced the motility and invasive ability of these cells both *in vitro* and *in vivo* [54]. These findings indicate that βig-H3 can restrain the metastatic potential of cancer cells and thus support the tumor suppressor function of βig-H3. Stable βig-H3 knockdown mutants established from a mesothelial cell line, Met-5A, exhibited an elevated proliferation rate, enhanced plating efficiency, increased anchorage-independent growth, and a more active PI3K/AKT/mTOR signaling pathway [55]. These findings suggest that βig-H3 may repress mesothelioma tumorigenesis and progression by inhibiting the PI3K/AKT signaling pathway.

#### 3.2.2. Roles as Tumor Promoter

Although there is strong evidence that βig-H3 has a tumor suppressor function, there is also convincing data in the literature reporting a tumor-promoting role for βig-H3 (summarized in Table 2). High βig-H3 expression has been shown for various tumor tissues and cell lines [6,62–70] and in many cancers elevated expression also relates to more aggressive tumors [6,70,71]. Furthermore, several reports indicate that βig-H3 can mediate cancer cell invasion and metastasis as well as enhance cancer cell extravasation [71–74].

βig-H3 has been shown to mediate lymphatic endothelial migration and adhesion to ECM under low oxygen conditions [75]. These observations suggest that during hypoxia, which commonly occurs in tumors, βig-H3 may aid the metastatic process by promoting the adhesion to lymphatic endothelial cells. More recently βig-H3 has been shown to be highly expressed by mesenchymal stem cells derived from human adipose tissue and to stimulate proliferation and adhesion of the A459 human lung adenocarcinoma cell line [20]. Furthermore, βig-H3 observed at the invasion front of melanomas co-localized with fibrillar fibronectin/tenascin-C/periostin structures, suggesting an important role for βig-H3 in ECM deposition and invasive growth of melanoma cells [76]. siRNAs against βig-H3 transfected into human hepatocellular carcinoma cells showed that βig-H3 increases the invasive potential of those cells by regulating MMP-2 and -9 secretion [77]. Thus, due to its tumor promoting role βig-H3 is a promising therapeutic target.

#### 3.2.3. Role of βig-H3 in Ovarian Cancer

##### 3.2.3.1. Tumor Suppressive role of βig-H3 in Ovarian Cancer

One of the crucial steps in ovarian cancer metastasis involves the implantation of ovarian cancer cells onto the peritoneal lining. As the underlying molecular mechanisms have not been well characterized we have studied the interaction between ovarian cancer and peritoneal cells *in vitro*. The ECM protein βig-H3 was found to be differentially regulated in the secretome of peritoneal-ovarian cancer cell co-culture. We demonstrated that βig-H3 is abundantly expressed by peritoneal cells and can promote ovarian cancer cell motility, invasion, and adhesion to LP-9 peritoneal cells [61].

**Figure 2:**
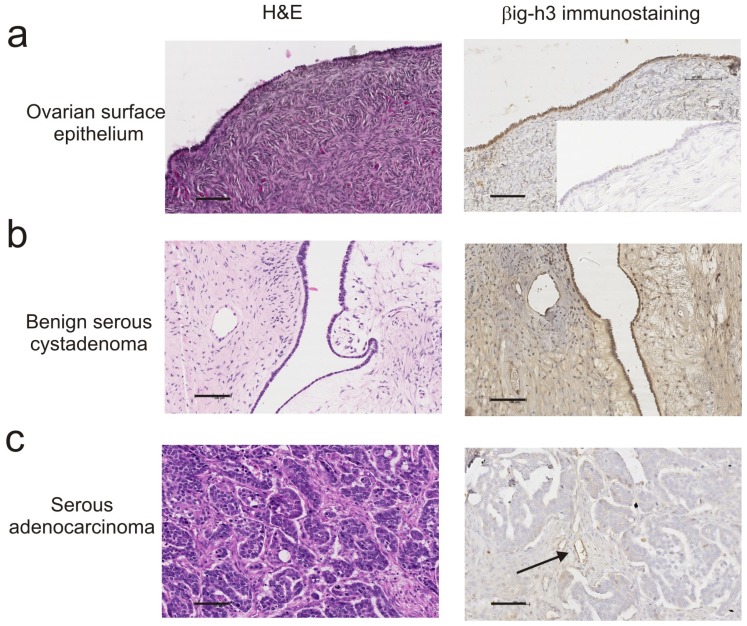
H & E and βig-H3 immunostaining of ovarian tissues. (**a**) Normal ovary surface epithelium; (**b**) Benign serous cystadenoma; (**c**) Serous ovarian carcinoma. Scale bar = 100 μm for all images. Immunostaining with polyclonal rabbit βig-H3 antibody (Santa Cruz Biotechnology) as described in [61].

Our recent studies investigating the role of βig-H3 in ovarian tumorigenesis have demonstrated low expression of βig-H3 in ovarian cancer cell lines and ovarian cancer tissue [61]. This is consistent with other studies demonstrating a down-regulation of βig-H3 in cancer cells and more recent studies demonstrating that the *TGFBI* gene is frequently hypermethylated in ovarian tumors [59,60]. Our data, showing high levels of βig-H3 immunostaining in normal ovarian surface epithelial cells (Figure 2a) and benign serous ovarian tumors (Figure 2b) but low βig-H3 immunostaining in human serous ovarian cancer cells (Figure 2c), suggest that βig-H3 is down-regulated during the process of ovarian cancer tumorigenesis [61]. Our findings, that high concentrations (>5 μg/mL) of βig-H3 can induce ovarian cancer cell death, also support an anti-tumorigenic role for βig-H3 [61]. The use of *TGFBI* methylation as novel epigenetic biomarker for discriminating ovarian cancer from non-cancer or borderline tumors [59] should be further explored.

##### 3.2.3.2. Pro-Tumorigenic Role of βig-H3 in Ovarian Cancer

In our recent study we have demonstrated that βig-H3 induces both motility and invasion of OVCAR-5 and SKOV-3 cells, but does not affect motility or invasion of OVCAR-3 ovarian cancer cells that are known to be less metastatic [61]. We have also shown that βig-H3 promotes attachment of OVCAR-5, SKOV-3, and OVCAR-3 to LP-9 peritoneal cells [61]. These findings suggest that βig-H3 may function in multiple ways to promote ovarian cancer metastasis and that the effects on motility may be independent of those on adhesion.

In our study, the effects of βig-H3 on OVCAR-5 cells were independent of the βig-H3 RGD integrin binding motif (amino acids 642–644), since treatment with ERGDEL peptide did not block the ability of βig-H3 to promote ovarian cancer cell motility, invasion, or adhesion to peritoneal cells. Our data suggests that βig-H3 activity on OVCAR-5 cells is mediated by other sites in the βig-H3 molecule other than the RGD motif, which may include the EPDIM and NKDIL motifs as well as the sequence spanning the YH18 motif.

##### 3.2.3.3. βig-H3 Processing by Ovarian Cancer Peritoneal Interactions

We have shown that βig-H3 cleavage in the ovarian cancer-peritoneal cell co-culture occurs between amino acid residues 27–76 in the *N*-terminus and amino acid residues 626–657 in the *C*-terminal domain [61]. Although the functional role of the *N*-terminal βig-H3 domain has not been well studied, the *C*-terminus has several integrin binding motifs including the RGD, YH18, and EPDIM sequences. βig-H3 fragments including the EPDIM and the RGD motif, have recently been shown to promote apoptosis of osteosarcoma cells [57]. A truncated βig-H3 lacking the EPDIM but not the RGD motif failed to induce apoptosis in this cell type [57].

Whilst it is not known whether the *C*-terminal processed βig-H3 in the secretome of the ovarian cancer-peritoneal co-culture retains its RGD sequence at amino acid 642–644, the EPDIM motif at amino acid 617–621 is maintained in the *C*-terminal processed βig-H3. Crystal structure of the FAS-1 domains (Drosphilia TGFBI/βig-H3 homologue) has identified a novel fold domain consisting of a seven-stranded β-wedge and a number of α-helices in the 3rd and 4th FAS-1 domains [88]. The EPDIM motif maps to a conserved kink in the β6 strand of the fourth βig-H3 FAS-1 domain and is predicted to be buried within the domain protein core [88]. βig-H3 processing by proteases, including plasmin between amino acids 626–655 may expose the EPDIM motif (amino acids 617–621) site for integrin interactions and may promote the integrin binding activity on the surface of the peritoneum [89–91] with ovarian cancer cells [92,93] and increase ovarian cancer metastatic behavior.

Interestingly, βig-H3 processing was only observed when ovarian cancer cells and peritoneal cells were in direct physical contact in culture, or when the cells shared the same growth media in the co-culture system [61]. βig-H3 processing did not occur when conditioned media from peritoneal cells was added to cultured ovarian cancer cell lines, or when conditioned media from ovarian cancer cells was added to the cultured peritoneal cells. This indicates that βig-H3 processing is not mediated by a simple up-regulation of ovarian cancer cell derived proteases but requires multiple levels of cross-talk between both ovarian cancer and peritoneal cells. A similar paracrine effect was previously reported for endometrial cancer epithelium–stroma cell co-cultures, where hepatic growth factor secreted by the stromal cells acted on the endometrial cancer cells by inducing the cleavage of MMPs pro-forms to mature active forms [94]. Our findings suggest, however, that cleavage of βig-H3 in the ovarian cancer and peritoneal cell co-culture is not MMP mediated as the broad spectrum MMP inhibitor, GM6001, failed to inhibit βig-H3 processing. Instead, we found that the protease plasmin cleaved βig-H3 in the same region as observed in the ovarian cancer-peritoneal cell co-culture and that this could be inhibited by a cocktail of protease inhibitors, including serine protease inhibitors. We demonstrated that plasmin activity was increased in the conditioned medium of co-cultured OVCAR-5 and LP-9 cells, whilst no plasmin activity could be detected in the conditioned medium collected from those cells cultured alone [61]. These findings add to our understanding of the interaction between ovarian cancer and peritoneal cells and suggest that increased plasmin production and βig-H3 cleavage may be early events in the process of ovarian cancer metastasis.

##### 3.2.3.4. βig-H3 as a Predictor of Therapy Response

The level of βig-H3 in ovarian cancer tissue has been shown to be a predictive marker of response to treatment with the aromatase inhibitor letrozole [95] and the chemotherapeutic drug paclitaxel [86]. The loss of βig-H3 induces a specific resistance to paclitaxel and is associated with mitotic spindle abnormalities in ovarian cancer cells [86]. Paclitaxel-resistant cells treated with recombinant βig-H3 protein show integrin-dependent restoration of paclitaxel sensitivity via FAK- and Rho-dependent stabilization of microtubules [86]. More recent studies have also shown that the suppression of β3 integrin and βig-H3 increase the resistance of SKOV3 to paclitaxel [87]. A strong association between elevated βig-H3 expression and the response to chemotherapy has also been identified in lung cancer patients [96]. Lung cancer cells over-expressing βig-H3 displayed increased sensitivity to etoposide, paclitaxel, cisplatin, and gemcitabine. βig-H3-mediated induction of apoptosis occurred through its binding to αvβ3 integrin by proteolytic fragments of βig-H3 and not full length protein [96]. Together these data show that βig-H3 is also a potential therapeutic to improve response to chemotherapy in ovarian cancer patients.

## 4. Conclusions

Studies over the last 5 years have increased our understanding of the role of βig-H3 in cancer. However, there is conflicting data in the literature reporting that βig-H3 can have a tumor suppressive as well as a tumor promoting role in different cancer cells. These opposing effects of βig-H3 have been identified in several different laboratories and are unlikely to be due to biased observations. βig-H3 expression and function in cancer cells appears to be cell type specific and is affected by βig-H3 concentration but also by processing events by protease enzymes which can liberate integrin binding sites. As truncated forms of βig-H3 have been well documented to have differing functions it is likely that alterations in βig-H3 processing in different cell types is an important factor contributing to the disparate findings in literature. Our findings highlight the need for amino acid sequencing to confirm the presence of full length or truncated forms of βig-H3 [61]. The findings that siRNA *TGFBI* knockdown increased melanoma cell growth and invasion *in vitro* but greatly impaired subcutaneous tumor growth in nude mice highlights the importance of the tumor microenvironment for βig-H3 function [76]. Whether βig-H3 functions as a tumor suppressor or tumor promotor may also be dependent on interactions between other ECM proteins and specific integrin receptors present in the tumor microenvironment.

Our research demonstrating that βig-H3 is down-regulated in ovarian cancer and promotes ovarian cancer cell death supports a tumor suppressor role. However βig-H3 is abundantly expressed by peritoneal cells and can promote metastatic behavior of ovarian cancer cells. Consequently, in ovarian cancer, βig-H3 may act as a “double-edged sword”. The loss of βig-H3 promotes ovarian tumorigenesis, microtubule and chromosome instability and a more chemoresistant phenotype, however in the peritoneal microenvironment; βig-H3 produced by the peritoneal cells aids the metastatic process. Our ovarian cancer studies to date indicate that βig-H3 is a potential therapeutic target to inhibit ovarian cancer metastasis to the peritoneum. Further studies investigating therapeutic strategies to block βig-H3 action in ovarian cancer are therefore warranted. βig-H3 derived peptides could be used to both block ovarian cancer metastasis and enhance chemotherapy response.

## Figures and Tables

**Table 1:** Studies reporting a tumor suppressor role for βig-H3.

Cell Type	Observation	References
CHO cells	βig-H3 inhibits cell attachment *in vitro* and suppresses the growth of CHO tumor cells in nude mice	[48]
	RGD peptides released from βig-H3 mediate apoptosis of CHO tumor cells	[49]
HeLa cells	RGD peptides released from βig-H3 mediate apoptosis of HeLa tumor cells	[49]
Bronchial epithelial cells	βig-H3 overexpression suppresses tumorigenicity in radiation-induced tumorigenic human bronchial epithelial cells	[50]
	Loss of βig-H3 expression is associated with the tumorigenic phenotype in asbestos-treated bronchial epithelial cells	[51]
	βig-H3 gene down-regulation is involved in heavy-ion radiation-induced tumorigenesis of human bronchial epithelial cells	[52]
Lung adenocarcinoma	Loss of βig-H3 protein is frequent in primary lung carcinoma and related to tumorigenic phenotype in lung cancer cells	[53]
	Promoter methylation contributes to promoter silencing of the βig-H3 gene in human lung cancer cells	[47]
	βig-H3 is down-regulated in radiation-induced thymic lymphoma model in BALB/c mice	[5]
	βig-H3 overexpression in H522 lung carcinoma cells reduces motility *in vitro* and metastasis *in vivo*	[54]
	RGD βig-H3 peptides mediate apoptosis of H1299 lung carcinoma cells	[49]
Mesothelioma cell lines	βig-H3 knockdown increases proliferation and anchorage independent growth of mesothelioma cell lines	[55]
Breast carcinoma	βig-H3 protein expression is reduced in *in situ* ductal carcinoma and breast carcinoma tissues, compared to benign tissues	[54]
	βig-H3 overexpression in MCF-7 cells reduces motility *in vitro* and metastasis *in vivo*	[54]
Neuroblastoma	βig-H3 significantly reduces proliferation and invasion of neuroblastoma cell *in vitro* and *in vivo*	[2,56]
Osteosarcoma	*C*-terminal fragment of βig-H3 is required for apoptosis in human osteosarcoma cells	[57]
Hepatoma	RGD βig-H3 peptides mediate apoptosis of Hep3B hepatoma cells	[47]
Knockout mice	βig-H3 knockout mice are prone to spontaneous tumors	[58]
Ovarian carcinoma	βig-H3 silencing and promoter hypermethylation is a frequent occurrence in ovarian cancer cell lines and ovarian cancer tissues	[59,60]
	βig-H3 is down-regulated in serous ovarian carcinoma and borderline serous ovarian tumors	[61]
	βig-H3 induces apotosis in serous ovarian carcinoma cell lines	[61]

**Table 2:** Studies reporting a tumor-promoting role for βig-H3.

Cell type	Observation	References
Lung adenocarcinoma	βig-H3 is overexpressed in lung cancer	[6]
	Recombinant βig-H3 stimulates proliferation and cell adhesion of A549 cells	[20]
Oesophageal adenocarcinoma	βig-H3 is up-regulated in oesophageal adenocarcinoma and esophageal squamous cell carcinoma tissues and cell lines tissue	[65,78,79]
Pancreatic cancer	βig-H3 expression is increased in pancreatic cancer cell lines and tissues	[68,80]
Oral squamous cell carcinoma	βig-H3 expression is increased in oral squamous cell carcinoma tissues	[81]
Brain tumors	βig-H3 promotes cell adhesion of human astrocytoma cells *in vitro* via interactions with α6β4 integrin	[72]
	βig-H3 expression is elevated in glioblastoma multiforme tissues	[82]
	Knockdown of βig-H3 inhibits glioma cell invasion and MMP secretion	[83]
Hepatocellular carcinoma	βig-H3 knockdown reduced invasion of 7721 cells	[73]
	βig-H3 interacts with α3β1 integrin to promote adhesion and invasion of 7721 cells	[74]
Colon carcinoma	βig-H3 expression is elevated in human colon carcinoma tissues	[64,84]
	Overexpression of βig-H3 promotes extravasation and enhances metastasis of colon cancer cells	[71]
Renal cell carcinoma	βig-H3 is up-regulated in clear cell renal carcinoma	[63,64]
	βig-H3 expression is increased in metastastic renal cell carcinoma	[85]
Ovarian carcinoma	βig-H3 suppression leads to a chemoresistant phenotype	[86,87]
	Recombinant βig-H3 promotes motility and invasion of OVCAR-5 and SKOV3 cells	[61]
	Recombinant βig-H3 promotes adhesion of OVCAR-3, OVCAR-5 and SKOV3 cells	[61]
